# Deep neural network prediction of modified stepped double-slope solar still with a cotton wick and cobalt oxide nanofluid

**DOI:** 10.1007/s11356-022-21850-2

**Published:** 2022-07-23

**Authors:** Swellam Wafa Sharshir, Ahmed Elhelow, Ahmed Kabeel, Aboul Ella Hassanien, Abd Elnaby Kabeel, Mostafa Elhosseini

**Affiliations:** 1 Mechanical Engineering Department, Faculty of Engineering, Kafrelsheikh University, Kafrelsheikh, Egypt; 2 Computers and Control Systems Engineering Department, Faculty of Engineering, Mansoura University, 35516, Mansoura, Egypt; 3 Electronics and Communication Engineering Department, Higher Institute of Engineering and Technology, New Damietta, Egypt; 4 Faculty of Computers & Information, Information Technology Department, Cairo University, Cairo, Egypt; 5 Faculty of Computers & AI, Scientific Research Group in Egypt (SRGE), Cairo University, Cairo, Egypt; 6 Mechanical Power Engineering Department, Faculty of Engineering, Tanta University, Tanta, Egypt; 7 Faculty of Engineering, Delta University for Science and Technology, Gamasa, Egypt; 8 College of Computer Science and Engineering, Taibah University, 46421, Yanbu, Saudi Arabia

**Keywords:** Solar still, Support vector regressor, Decision tree regressor, Neural network, Deep neural network

## Abstract

This research work intends to enhance the stepped double-slope solar still performance through an experimental assessment of combining linen wicks and cobalt oxide nanoparticles to the stepped double-slope solar still to improve the water evaporation and water production. The results illustrated that the cotton wicks and cobalt oxide (Co_3_O_4_) nanofluid with 1wt% increased the hourly freshwater output (HP) and instantaneous thermal efficiency (ITE). On the other hand, this study compares four machine learning methods to create a prediction model of tubular solar still performance. The methods developed and compared are support vector regressor (SVR), decision tree regressor, neural network, and deep neural network based on experimental data. This problem is a multi-output prediction problem which is HP and ITE. The prediction performance for the SVR was the lowest, with 70 (ml/m^2^ h) mean absolute error (MAE) for HP and 4.5% for ITE. Decision tree regressor has a better prediction for HP with 33 (ml/m^2^ h) MAE and almost the same MAE for ITE. Neural network has a better prediction for HP with 28 (ml/m^2^ h) MAE and a bit worse prediction for ITE with 5.7%. The best model used the deep neural network with 1.94 (ml/m^2^ h) MAE for HP and 0.67% MAE for ITE.

## Introduction

In the last decades, freshwater supplies have become seriously insufficient because of the excessive use and the increasing pollution of natural water resources (Elkadeem et al. 2021; Kotb et al. 2021). Also, the global drinking water demand increases because of population density and industrial growth, but the amount of freshwater is fixed (Shannon et al. 2010). Therefore, improving the performance of the cleaning water technologies to produce freshwater has become crucial for the twenty-first century (Elimelech 2006; Elmaadawy et al. 2020). There has been considerable effort made worldwide to prevent this issue while retaining the limited drinking water supply and converting the huge quantities of non-potable water obtained through various desalination methods into potable water. Recently, solar stills (SSs) have become one of the best efficient ways and solutions used for solar desalination to get fresh water in arid regions and have large advantages such as simplicity in contraction and eco-friendly. However, the SS still faces a problem due to its low freshwater production (Sharshir et al. 2017a).

Researchers have focused on different modifications to enhance the performance of SSs (Arunkumar et al. 2019; Sharshir et al. 2017a). Several design improvements were proposed and examined in order to improve the SS performance such as flat SS (Peng et al. 2021a), pyramid SS (Nayi and Modi 2018; Sharshir et al. 2020a), inclined SS (Kalidasa Murugavel et al. 2013; Kaviti et al. 2016), tubular SS (Kabeel et al. 2019b; Sharshir et al. 2019a; Wang et al. 2021), multi-basin (Shanazari and Kalbasi 2018), double-slope SS with rubber scrapers (Elsheikh and Abd Elaziz 2018), trapezoidal pyramid SS(Sharshir et al. 2022c), double-slope SS (Elmaadawy et al. 2021; Kandeal et al. 2021a, b; Raj Kamal et al. 2021; Sharshir et al. 2020e; Tuly et al. 2021), and stepped SS (Alaudeen et al. 2014; Kabeel et al. 2015). On the other hand, the most widely used methods for solar still modifications, nanofluids (Elsheikh et al. 2018; Sharshir et al. 2017c), wick material (Sharshir et al. 2020c; Sharshir et al. 2022a; Sharshir et al. 2020f; Sharshir et al. 2021d) nano-coating (Thakur et al. 2022), thin film evaporation (Elsheikh et al. 2019b; Peng et al. 2018, 2021b; Sharshir et al. 2022a), phase change materials (AbuShanab et al. 2022; Al-Harahsheh et al. 2022, 2018; Javadi et al. 2020; Shalaby et al. 2016), basin water depth (Khalifa and Hamood 2009; Muthu Manokar et al. 2020; Phadatare and Verma 2007), hydrogel materials (Sharshir et al. 2020b), v-corrugated aluminum basin (Abdelaziz et al. 2021a), carbonized wood with nano (Sharshir et al. 2022b) reflectors (Elmaadawy et al. 2020), energy storage (El-Shafai et al. 2022; Sharshir et al. 2021a, 2019b; Thakur et al. 2021b), nano-based mushrooms (Sharshir et al. 2021b), heat localization materials (Sharshir et al. 2020e), cover cooling (Elsheikh et al. 2021; Sharshir et al. 2016a, 2022a, 2017c), evacuated tubes (Mevada et al. 2022), graphene nano-ratchet (Ding et al. 2017), solar collector (Abu-Arabi et al. 2020, 2018; Sharshir et al. 2019c; Thakur et al. 2021c), porous absorber (Abdelaziz et al. 2021d), and hybrid systems (ABDELAZIZ et al. 2021b; Abdelaziz et al. 2021e; Sharshir et al. 2016b; Sharshir et al. 2016c).

Pyramid still coated with TiO_2_ nano black paint (Kabeel et al. 2019a), inserting internal or external condensers (El-Bahi and Inan 1999), inserting internal or external reflectors, cooling the glass cover (Sharshir et al. 2017c), using silica nanocomposites which are fumed in black paint (Sathyamurthy et al. 2020), as a porous absorber, using activated carbon (Abdelaziz et al. 2021c), using nanomaterials (Sharshir et al. 2020d; Sharshir et al. 2018), the use of phase change materials or gravels (Sharshir et al. 2017b), humidification-dehumidification solar still (Sharshir et al. 2016b, 2016c), atomizer with ultrasonic waves (El-Said and Abdelaziz 2020), the use of airing multifunctional textile (Peng et al. 2020), chips made of wick metal (Sharshir et al. 2020f), and absorber made of graphene oxide (Thakur et al. 2021a). SS integrated with nanoparticles (Elsheikh et al. 2019b, 2018; Sharshir et al. 2018), energy storage (Yousef and Hassan 2019), sponges (Sellami et al. 2017), wick (Pal et al. 2018), painted the still basin with nanomaterials (Kabeel et al. 2019a), and so on.

It is illustrated that the saline water depth in the SS basin inversely affects the freshwater yield. The water depth control to maintain it at a minimum value in the SS is a cumbersome problem. Many modifications were proposed to achieve this purpose, such as cubes made of sponge material in the water basin (Sharshir et al. 2016d). The tentative performance of a SS combined with a small stratum of thermal material storage beneath the absorber plate to produce freshwater during sunset was investigated (El-Sebaii et al. 2009).

Using the wick materials which act through the capillary action improved the evaporation rate. This is because it does not need high energy to heat the whole water; on the other hand, solar irradiance focuses on the water in thin wick material. In addition, using these wick layers solved the problem of the dry spots appearing due to the decrease in water depth, thus increasing the evaporation rate. Also, Murugavel and Srithar (2011) employed several wick materials such as waste cotton pieces, coir mate, sponge sheet, and light cotton cloth to increase the area of evaporation to enhance the SS production. The light black cotton cloth proved to be the most effective wick material. Alaian et al. (2016) used a pin-finned wick to increase the solar still’s water productivity. The daily output of water rose by 13% over the traditional solar, still demonstrating the influence of the solar reflector. Hansen et al. (2015) examined how several wick materials (wicking water coral fleece fabric and wood pulp paper wick) affected various plate absorbers (stepped absorber, flat absorber, and stepped absorber with wire mesh). The result showed that when utilizing a wire mesh-stepped absorber with water coral fleece, the highest water output was 4.28 L/day.

However, in an experimental and simulation study, other researchers improved water evaporation by adding various thin-film nanostructures and heat localization with water desalination (Peng et al. 2020; Sharshir et al. 2020d). It was found that, at 1000 W/m^2^ irradiance, the thermal efficiency was 78% (Ghasemi et al. 2014). The effects of expanded carbon foam and graphite on evaporation rate and efficiency were explored using a double-layer structure, which achieved 67% at 1000 W/m^2^. Otherwise, employing a polystyrene and graphene oxide double-layer structure increased the efficiency of evaporation by 80% (Li et al. 2016). However, it should be noted that more investigations are needed for the real application of this material, and its usage remains very difficult.

Metallic surfaces are vital for enhancing the process of heat transfer in solar stills. On the other hand, nanofluid may promote corrosion and erosion on a metallic surface by both physical and chemical mechanisms (Celata et al. 2014). The metallic surface will be consumed noticeably and rapidly when the typical chemical corrosion range is available. The fluid’s characteristics fall in it—even if within a limited time interval (Bubbico et al. 2015). At the same time, the collision between the metallic surface and particles when using nanofluid will erode the bent pipes (Shamshirband et al. 2015; Shinde et al. 2019). So that, before using nanofluid in desalination systems, the erosion and corrosion must be examined to avoid any undesirable interactions between components (Celata et al. 2014). It is also useful to benefit from nanofluid’s advantage for decreasing corrosion and erosion by forming a compact protective film on the metallic surface (Sha et al. 2019). Proper system design and maintenance are necessary to reduce the effects of corrosion and erosion (Muthanna et al. 2019).

Nanofluid stability and pressure drop are other problems besides erosion and corrosion phenomena. Utilizing nanofluids in solar desalination is still one of the biggest long-term nanofluids’ issues that require more research. Poor stability causes particles to accumulate and settle in addition to chemical dissolution, and thus the nanofluids fail (Sezer et al. 2019). In addition, there will be a high accumulation of nanoparticles for passive devices, especially at high temperatures, due to the lack of a pump to circulate and move the nanoscale fluids (Taylor et al. 2012). When using nanoparticles, pressure drop and passive solar still pumping problems will arise. On the other hand, as the concentration of nanoscale fluids increased, the pressure drop under the turbulent system increased accordingly (Duangthongsuk and Wongwises 2010). Also, the rise in the pressure drop will necessarily raise the system operation cost.

Because of the need for precise and dependable modeling of solar energy systems, ANN models have been used to replace conventional models (Delfani et al. 2019; Elsheikh et al. 2019a; Kumar et al. 2019; Motahar and Bagheri-Esfeh 2020; Nasruddin et al. 2018). They have been successfully utilized because of their ability to deal with the extreme uncertainty of these data. ANN has been described as a robust tool for modeling various engineering systems (Babikir et al. 2019; Essa et al. 2020; Shehabeldeen et al. 2020, 2019). Santos et al. (2012) predicted the distillate production of a conventional SS using ANN and the local weather data in Las Vegas and the USA, using different parameters such as solar radiation, average daily air velocity, and the air direction, cloud cover, and air temperature. The results illustrated that with enough input data, the prediction of the SS performance using the ANN method works very effectively at various condition parameters. Hamdan et al. (2013) conducted three different ANN models, namely, nonlinear autoregressive exogenous, feed-forward ANN, and Elman NN, to predict a triple SS performance. The experiments were conducted under weather conditions in Jordan. The input data were air temperature; solar radiation time; glazier temperature; the water temperature in the upper, middle, and lower basins; freshwater output; and plate temperature. Results illustrated that the feed-forward ANN is a good tool for getting the wanted performance (Mashaly and Alazba 2017). The inclined SS immediate efficiency, water yield, and operating recovery ratio were predicted using an ANN model. The findings showed that the ANN model was accurate and effective in predicting SS performance with minor mistakes. Most of the operational and meteorological parameters that affect evaporation and condensation processes in the desalination unit were not addressed according to Hamdan et al. (2013) and Santos et al. (2012). Moreover, the contribution of all components is not determined in the modeling process.

Because of the need for a reliable and accurate simulation of the wick SS productivity every hour, ANN models were used. ANN can be trained using a few experimental data and then study the input and output nonlinear relationship. Once the training process is accomplished, ANN can predict the productivity for any inputs (conditions) that it has not seen before without involving in conducting more experiments or solving complicated mathematical models. Despite the generalization capabilities and robustness of ANN, the traditional ANN still faces some limitations related to the determination of the ANN model parameters. As the determination of ANN model parameters has a significant effect on the ANN performance, many methods have been reported in the literature to determine these parameters, such as backpropagation (Chen 1990), conjugate gradient (Saini and Soni 2002a), and Quasi-Newton’s method (Saini and Soni 2002b). However, these traditional methods are easy to stick to local solutions, which affects the final quality of the ANN.

According to the literature analysis, multiple designs with different amendments were carried out to improve solar performance further. Several approaches need extra components such as condensers, collectors, and reflectors to increase the area exposed to insolation. Although the yield of fresh water was greater, this led to high costs and poor efficiency. Very little research examines the influence of carbon black cobalt oxide (Co_3_O_4_) on SDSS performance paired with cotton wicks. Furthermore, four different machine learning models were used, i.e., support vector regressor (SVR), decision tree regressor, neural network, and deep neural network. The results showed that the neural network gave the worst results, especially for validation and test data, which means it failed to generalize. Hence, the neural network is almost out of comparison.

To conclude and summarize the related work, one may suppose that ANN gives the best prediction accuracy that can be achieved. However, no single study was investigated using SVM, decision tree, or deep neural network to predict the stepped double-slope solar. On the other hand, ANN can achieve good accuracy for the two outputs of our system simultaneously. That is why we investigated using more recent and stronger machine learning models, namely SVM, decision tree, and deep neural network.

The main contribution of this research paper is achieving a very high accuracy for the prediction of the two outputs of the stepped double-slope solar still system, namely the HP and the ITE, simultaneously. Moreover, we executed a comparative and statistical analysis of the deep learning model against other states of the art ML methods. Furthermore, we studied the importance and ranking of features w.r.t the outputs’ prediction.

The organization of the research paper is as follows: the “Experimental setup” section covers the experimental setup of the stepped double-slope solar still. The “Instantaneous thermal efficiency calculation and error estimation methods” section covers definitions and formulas of error metrics. The “Methods” section briefly explains the ML methods used for the prediction and introduces the idea of feature selection. The “Results and discussions” section covers the results for each of the previously explained methods in the “Methods” section and compares the results of these methods. The “Results and discussions” section also covers the feature selection results. Finally, the “Conclusions” section covers the conclusion and determines the best model for predicting the stepped double-slope solar still system.

## Experimental setup

**Fig. 1:**
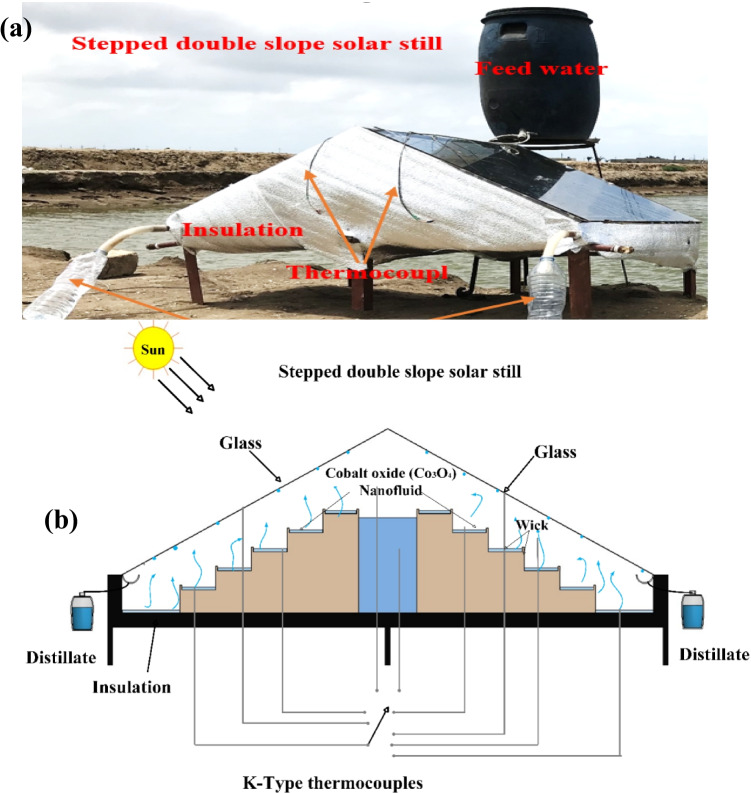
Experimental setup. (**a**) Photograph, and (**b**) schematic

The experiment was carried out in the open air of Al-Burullus City in Kafrelsheikh, Egypt. (latitude 31.07° N and longitude 30.57° E) from 9 am to 5 pm (GMT + 2) during 5 days in May, 2020 (10th, 15th, 25th, 28th, and 30th) and 4 days in June, 2020 (5th, 12th, 18th, and 25th). All experiments were conducted with saline water from Burullus Lake. The sample had a total dissolved solid of 1400 ppm, and a pH value of 8.7 in the northern part of the Nile River Delta, near the Mediterranean Sea, Kafrelsheikh, Egypt. Figure 1(a, b) illustrates pictorial and schematic views of the experimental setup, respectively.

**Fig. 2:**
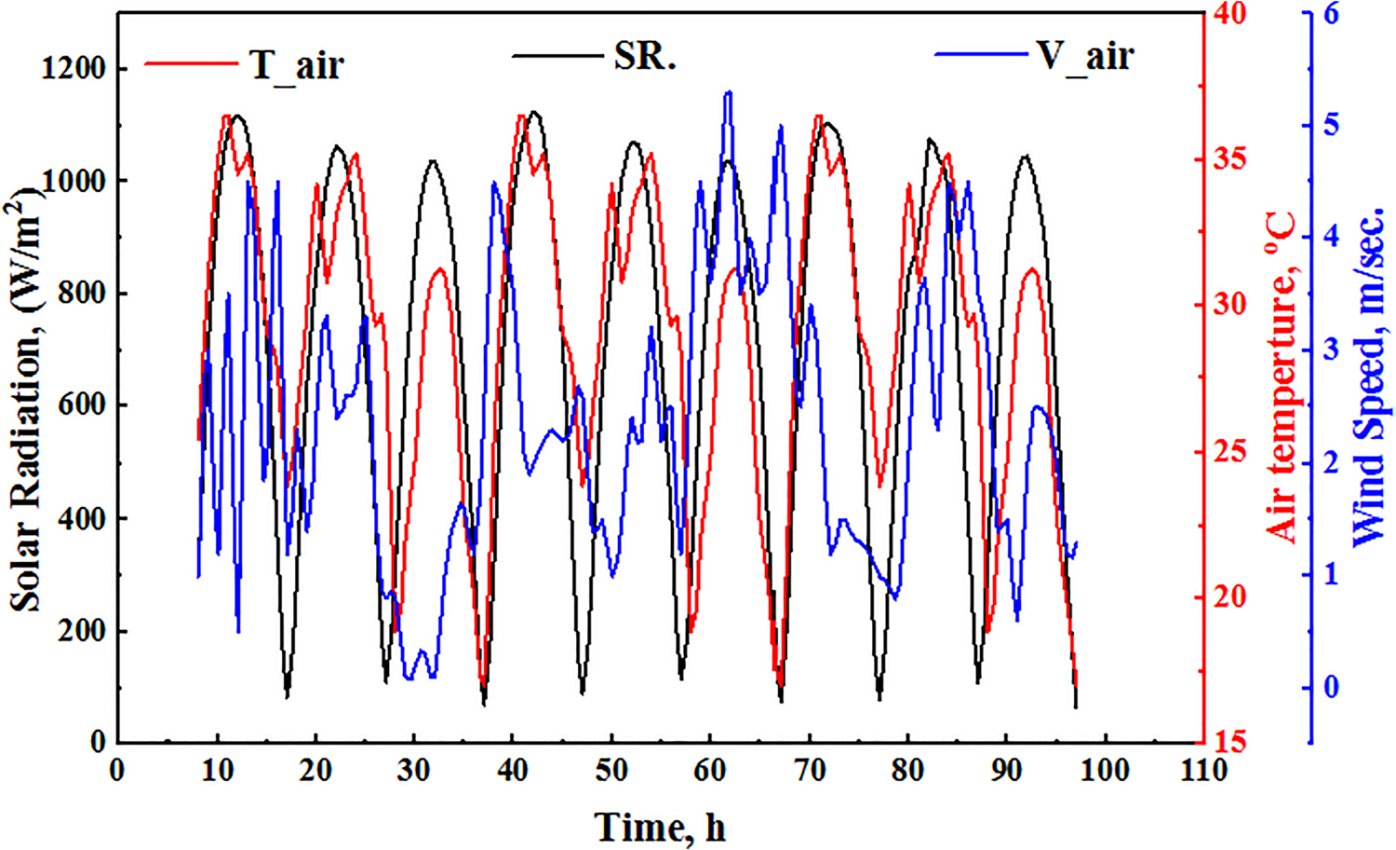
Illustration of the training dependent input data variables SR, ambient temperature, air velocity

The setup of the experiment consisted of a stepped double-slope solar still (SDSSS). The still was made of a sheet of iron with a thickness of 1.5 mm. All inner surfaces were painted black to absorb as much solar energy as possible. All outer surfaces (basin and sidewalls) of solar stills were suitably insulated to reduce heat losses to the ambient. The space bounded by the backside of steps in SDSSS, the wall of the water basin located inside SDSSS, and the frame of SDSSS were filled with wood shavings, as shown in Fig. 2. Wood shavings were an insulation material that prevents heat transfer from the steps’ bottom surfaces to space. A transparent glass cover of 3-mm thickness and inclined with ~ 30° horizontally was used to cover the SDSSS (almost the latitude angle of the location of the experiment). The saline water depth in the SSs was conserved at 1 cm. The projected area of SDSSS steps was 0.975 m^2^ (0.75 m × 1.3 m), and the area of the glass cover was 0.56 m^2^ (0.75 m × 0.75 m) for each side. The SDSSS was oriented in the East–West direction to absorb the maximum possible insolation. The nanofluid was obtained by mixing Co_3_O_4_ with the feed water at 1 wt%. The utilized Co_3_O_4_ nanoparticles had average grain size of 14 nm. The thermophysical properties of the Co_3_O_4_ particles are given in Table 1 and the cotton black wick was used.

**Table 1:** Thermophysical properties of Co_3_O_4_ particles

Property	Co_3_O_4_
Density, (kg/m^3^)	611
Melting point, (°C)	895
Thermal conductivity, (W/m·K)	31
Specific heat, (kJ/kg K)	0.81
Thermal expansion coefficient, (1/k)	0.809

The experimental setup was equipped with a suitable measuring instrument to record several parameters’ variations at hourly intervals, such as temperatures of vapor, water inside the stills, glass covers, and the ambient air. K-type thermocouples (the range was from − 50 to 180 °C, and the accuracy was ± 1 °C) were used to evaluate these parameters which are connected to a digital temperature indicator (Manufacturer TES Electrical Electronic Corp., Model 305P). Solar irradiance in the East and West directions was measured by the solar meter of Manufacturer TES Electrical Electronic Corp., model TES-1333R (the range was from 0 to 2000 W/m^2^, and the accuracy was ± 10 W/m^2^). The wind velocity was measured using a vane-type digital anemometer manufacturer BENETECH, Model GM816 (the range was from 0.1 to 30 m/s, and the accuracy was ± 0.1 m/s). Freshwater productivity was measured using a graded cylinder, and the accuracy was ± 2 ml. Uncertainty in length, width, diameter, and thickness measurements were ± 0.5 mm.

The uncertainty of an estimated value derived from the uncertainty of observed parameters is known as a propagation of uncertainty. To calculate this function, the following equation was used (Cohen 1998; Dhivagar and Mohanraj 2021):

1 $$w_{x} = \sqrt {\left( {\frac{\partial X}{{\partial x_{1} }}} \right)^{2} wx_{1}^{2} + \left( {\frac{\partial X}{{\partial x_{2} }}} \right)^{2} wx_{2}^{2} + ............ + \left( {\frac{\partial X}{{\partial x_{n} }}} \right)^{2} wx_{n}^{2} }$$

where $w$ is the measured parameter uncertainty, $x_{n}$ is the parameter of interest, and $w_{x}$ is the uncertainty propagation for $X$ value.

## Instantaneous thermal efficiency calculation and error estimation methods

The immediate thermal efficiency is one of the most essential factors in estimating the distiller’s performance (Sharshir et al. 2017a). It represents the useful energy $\left(\frac{{\dot{m}}_{d}}{3600} \times {h}_{fg}\right)$, divided by the energy input $\left(I\left(t\right)\times {A}_{s}\right)$.

Instantaneous thermal efficiency ${\eta }_{\mathrm{ite}}$ calculated as follows:

2 $${\eta }_{ite}=\frac{\frac{{\dot{m}}_{d}}{3600} \times {h}_{fg}}{I\left(t\right)\times {A}_{s}}\times 100;\;(\%)$$

where ${\dot{m}}_{d}$ is the freshwater production (kg/h); ${A}_{s}$ is the distiller basin area (m^2^); *I*(*t*) is the solar intensity (W/m.^2^); and ${h}_{fg}$ is the latent heat (J/kg) calculated as (Kabeel et al. 2018)

3 $${\mathrm{h}}_{fg}={10}^{3}\times \left[2501.9-2.40706\;{T}_{\mathrm{w}}+1.192217\times {10}^{-3}{{\;T}_{\mathrm{w}}}^{2}-1.5863\times {10}^{-5} {{\;T}_{\mathrm{w}}}^{3}\right]$$

This section provides the verbal definitions, and mathematical formulas for mean absolute error (MAE), root mean square error (RMSE), mean relative error (MRE), and R-squared score.

MAE is the sum of the absolute values of errors for all samples divided by the number of these samples.

4 $$\mathrm{MAE}=\frac{{\sum }_{i=1}^{n}\left|{e}_{i}\right|}{n}$$

RMSE is the root of the division of the sum of the errors squared of all samples by the number of these samples.

5 $$\mathrm{RMSE}=\sqrt{\frac{{\sum }_{i=1}^{n}{\left({e}_{i}\right)}^{2}}{n}}$$

MRE is the sum of the division of the absolute error by the actual measurement for all samples divided by the number of these samples.

6 $$\mathrm{MRE}=\frac{{\sum }_{i=1}^{n}\left|{e}_{i}\right|/{y}_{i}}{n}$$

The R-squared score is the goodness of the model’s fit such that one is the superior value. R-squared score equals one minus the division of the residual sum of squares by the total sum of squares. The residual sum of squares, SS_res_, is the sum of the square of the difference between the actual value and the predicted value for all samples. The total sum of squares, SS_tot_, is the sum of the square of the difference between the actual value and the average of all the actual values for all samples.

For SVR, decision tree, and deep neural network, the *sklearn* library in python language has been used to obtain R-squared score, RMSE, and MAE. However, MRE has been calculated manually using a simple for loop. On the other hand, for neural network, RMSE and MAE has been obtained using RMS and MAE MATLAB functions. However, R-squared and MRE have been calculated manually using simple MATLAB code that implements the equations of each score.

$$\mathrm{R}-\mathrm{squared }=1-\frac{{\mathrm{SS}}_{\mathrm{res}}}{{\mathrm{SS}}_{\mathrm{tot}}} , {\mathrm{SS}}_{\mathrm{res}}=\sum\nolimits_{i=1}^{n}{\left({e}_{i}\right)}^{2} , {\mathrm{SS}}_{\mathrm{tot}}=\sum\nolimits_{i=1}^{n}{\left({y}_{i}-{y}_{\mathrm{avg}}\right)}^{2}$$

## Methods

### Support vector regression

Since our data may have outliers due to faults in measurement equipment, we chose to build our first model using SVR.

SVR is a supervised machine learning algorithm that works on finding a line, hyperplane, or curve when using kernels such that all data points exist within the decision boundaries of the SVR (Ahmad et al. 2018). Hence, we use a road or street instead of a line or curve. This road can be linear, polynomial, or nonlinear, depending on whether we use kernels. We can also control the width of this road using hyperparameter C. Wider road means that some predictions are accepted despite some errors. On the other hand, the narrower road leads us to regular regression (Ma and Guo 2014). Support vector machines generally have a good reputation for dealing with data outliers (Géron 2019). The python language was chosen since it has many reliable libraries in machine learning. We used the *sklearn* library within python since it offers a wide range of options regarding the hyper-parameters of the SVR (Parbat et al. 2020).

### Decision tree regressor

After that, we decided to test the decision tree regressor so that our model prioritizes the eight input features (Karax et al. 2019). The decision tree is a tree-like modeling method that can be used for classification and regression. After proper training, it can be reconfigured to be expressed in only a few conditional control statements. It is a white box machine learning technique (Ray 2019). Decision tree uses Gini impurity to determine which feature to check first, which helps to separate the most repeated class in its branch inside the tree (Daniya et al. 2020). Using the Gini impurity technique within the decision tree allows for determining the input features priorities that can be used to predict the desired output (Yuan et al. 2021). One node is pure with Gini impurity equals zero if all training data belonging to this node are from the same class. However, we must be careful about overfitting problems that are very common when using a decision tree (Suthaharan 2016).

Python language and *sklearn* library were chosen for the same reason as SVR. Besides very good documentation, *sklearn* provides a wide range of options and examples for the decision tree algorithm like SVR (Mohagaonkar et al. 2019).

### Artificial neural network

Neural networks are adaptive systems that can learn from given data to simulate the real model that generated these data (Jani et al. 2017). The neural network structure is based on interconnected neurons in layers to resemble the human brain (Delfani et al. 2019). The neural network is a set of connected input/output relationships with weight on each connection. The standard structure consists of one input layer, one output layer, and one or zero hidden layer [80]. The learning procedure is based on updating the weight of connections to decrease the error until convergence. Iteratively updating the weights leads to increased network performance.

We used ***nntool***, which is a part of the MATLAB Deep Learning toolbox in MATLAB software [81]. MATLAB ***nntool*** divided the 90 rows of data into 62 as training data, 14 as validation data, and 14 as testing data. More sophisticated machine learning models have been experimented with because our system has eight input features and two outputs to predict.

### Deep neural network

Deep neural network is an advanced topic of machine learning. Experiments admitted that deep neural network has more capabilities in the prediction process than any other method in machine learning (Ramsundar and Zadeh 2018). That is why our main prediction model is based on a deep neural network. The deep neural network is only a multi-layer neural network. It is based on the neural network method but with many hidden layers between input and output layers. A deep neural network can model complex nonlinear data (Shridhar 2017). Python language was chosen for the same reason as SVR and decision tree. Regarding deep neural network, the TensorFlow library with keras API was chosen since TensorFlow is an open-source library for deep learning algorithms, and keras provides the high-level API that simplifies the coding procedure (Gulli and Pal 2017). Keras supports all models of deep neural networks.

Our deep neural network model comprises a flatten layer, five dense layers with 50 neurons each, and an output layer with only two neurons because we have two outputs. For the training procedure, the Adam optimizer was used.

### Feature selection

In raw data, features are properties measurable with their corresponding class ownership information. Unfortunately, the curse of dimensionality limits current methods; too few subjects are available for training compared with large features. Furthermore, feature vectors of high dimension generally contain redundant or irrelevant information, which can lead to overfitting and reduced generalizability of the algorithm (Shi et al. 2022). The dataset is an important factor directly influencing machine learning performance(Turkoglu et al. 2022). According to researchers, machine learning relies more on clean data than on better algorithms. Training the model becomes more difficult with more features in the dataset. It worsens the model’s performance when unnecessary features are included in the dataset. The goal of feature selection is to eliminate these unnecessary features from the model before training to increase the model’s success. Many redundant or irrelevant features increase the computational burden (Tiwari and Chaturvedi 2022), resulting in the “curse of dimensionality.” Feature selection (FS) helps select the optimal classifier by choosing the most relevant features to decision-making (Qaraad et al. 2022). The number of possible solutions exponentially increases as the number of features increases in FS, which is an NP-hard combinatorial problem. Many different approaches have been put forward for feature selection in the literature. Feature selection methods fall into three main categories: (1) filters, (2) wrappers, and (3) hybrid or embedded methods (Hancer et al. 2022; Tiwari and Chaturvedi 2022). In conjunction with statistical data analysis metrics such as correlation and distance, filter-based methods such as principal component analysis, F-scores, and information gains identify subsets of features in the data (Qaraad et al. 2022). Despite their speed, the methods do not depend on the learning algorithm. Despite this, they neglect the importance of various dimensions when choosing the features to include in a subset. Wrapper-based algorithms, on the other hand, seek to find a near-optimal solution from an exponential set of possible solutions. In wrapper-based strategies, the subsets are identified based on the predictability of the classifiers. Filtering and wrapping are combined in different ways through hybrid methods. Their first step is to combine selected features with their learning algorithm. In addition, since the optimal feature set is not evaluated repeatedly, they are less expensive than wrapper methods (Tiwari and Chaturvedi 2022). Each input feature is given a priority value in this work using a random forest regressor as a wrapper method. Random forest regression was implemented using the Python package Sklearn.

### Computing environment

Regarding computing results for SVR, decision tree regressor, and deep neural network, Python language has been used and run on the Google Colaboratory service, Colab. Colab is a Jupyter notebook environment completely free of charge that stores users’ notebooks on Google Drive and runs in the cloud. Google Colab CPU service provides 12.68 GB of RAM and 107.72 GB of temporary disk storage. However, regarding the neural network model, MATLAB has been used and run on a laptop Dell Inspiron n5520 with Intel(R) Core (TM) i5-3210 M CPU @ 2.5 GHz, 8 GB RAM, and operating system Windows 10 Pro 64-bit.

## Results and discussions

Every hour from 9:00 to 17:00 (GMT + 2) throughout the day, the results were measured and repeated for 9 days. Figure 2 illustrates the sample of the experimental input variables data, which depends on the weather conditions and cannot be controlled, namely insolation, ambient temperature, inlet water temperature, and air velocity for the suggested model. Figure 2 demonstrates a sample of insolation data every hour; the mean insolation was 827.35 W/m^2^. Figure 2 demonstrates an example of ambient temperature; the mean value of the air temperature was 29.86 °C, respectively. Furthermore, Fig. 2 illustrates air velocity in meter/second; the mean air velocity was 2.36 m/s.

**Fig. 3:**
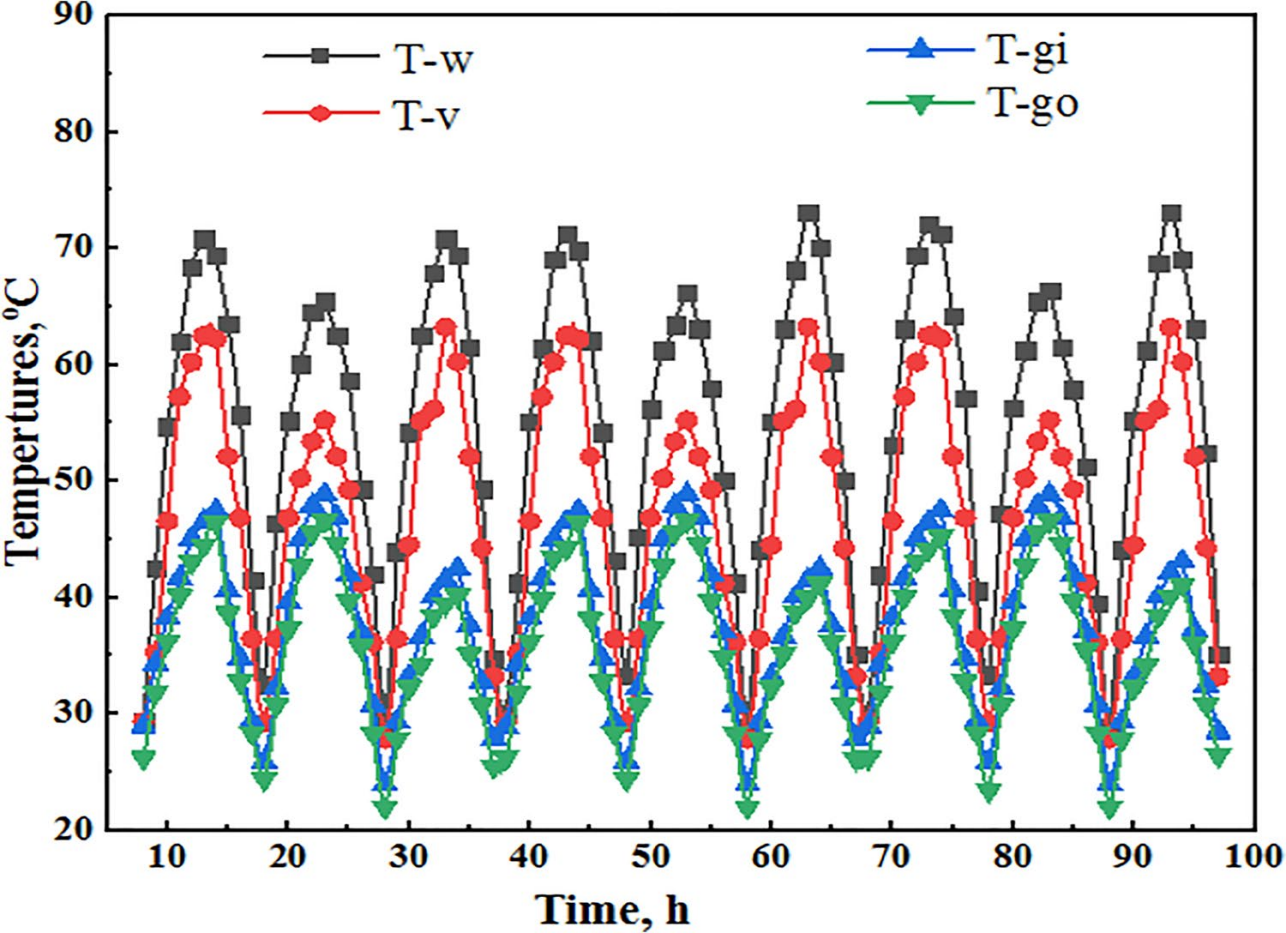
Samples of the dependent variables of SDSS were extracted from the training data set: (**a**) wick, (**b**) water, (**c**) vapor, and (**d**) glass temperatures for the proposed ANN and ANN-TSA model

Furthermore, Fig. 3 illustrates samples of the dependent variables used as inputs during the training process of the suggested model. Figure 3 shows the water temperature, glass temperature (in and out), and vapor temperature for SDSSS, respectively. The maximum and minimum water temperature was about 69 and 33 °C, respectively, while the average vapor temperature was about 54.61 °C. Also, the average glass inlet temperature was about 52.69 °C, and the average glass outlet temperature was about 46.73 °C. Furthermore, the comparison between the accumulated efficiency and freshwater production of present results with other available works is illustrated in Table 2.

**Table 2:** Comparison between the accumulated efficiency and freshwater production of the present results with other available works

Reference	Type of distiller and modification	Accumulated efficiency, %	Accumulated production, L/m^2^
**Present results**	Modified stepped double slope solar still with a cotton wick and cobalt oxide nanofluid	**38**	**4**
Sharshir et al. (2022c)	Single-slope distiller	33	3
Kandeal et al. (2021a, b)	Double-slope distiller with nano and energy storage	68	6.52
Pal et al. (2017)	Multi-wick in double-slope distiller	23.03	4.5
Shalaby et al. (2016)	Conventional pyramidal distiller with v-corrugated and energy storage	–	3.32
Sharshir et al. (2020e)	Wick with black carbon nanomaterials in stepped basin double-slope distiller	60.20	4.46
El-Sebaii et al. (2009)	Conventional distiller with energy storage	37.80	4.01
Kabeel et al. (2019a)	Nanocoating basin of pyramid distiller with TiO_2_	–	6.6
Wassouf et al. (2011)	Conventional pyramidal distiller	–	2.394
Sharshir et al. (2021c)	Conventional distiller with floating coal, wick, and black carbon	52.5	5.23
Sharshir et al. (2022c)	Trapezoidal pyramid distiller with hanging wick	45.10	3.97
Elmaadawy et al. (2021)	Double-slope distiller with wick, thermal storage, and nano additives	59.47	4.91

Furthermore, the experimental data were divided into training data and testing data using k-fold cross-validation such that the data was split into ninefold. Hence, each epoch contains nine runs. Each run, a different part of data, is considered testing data. At the end of the nine runs, each fold was considered testing data.

**Fig. 4:**
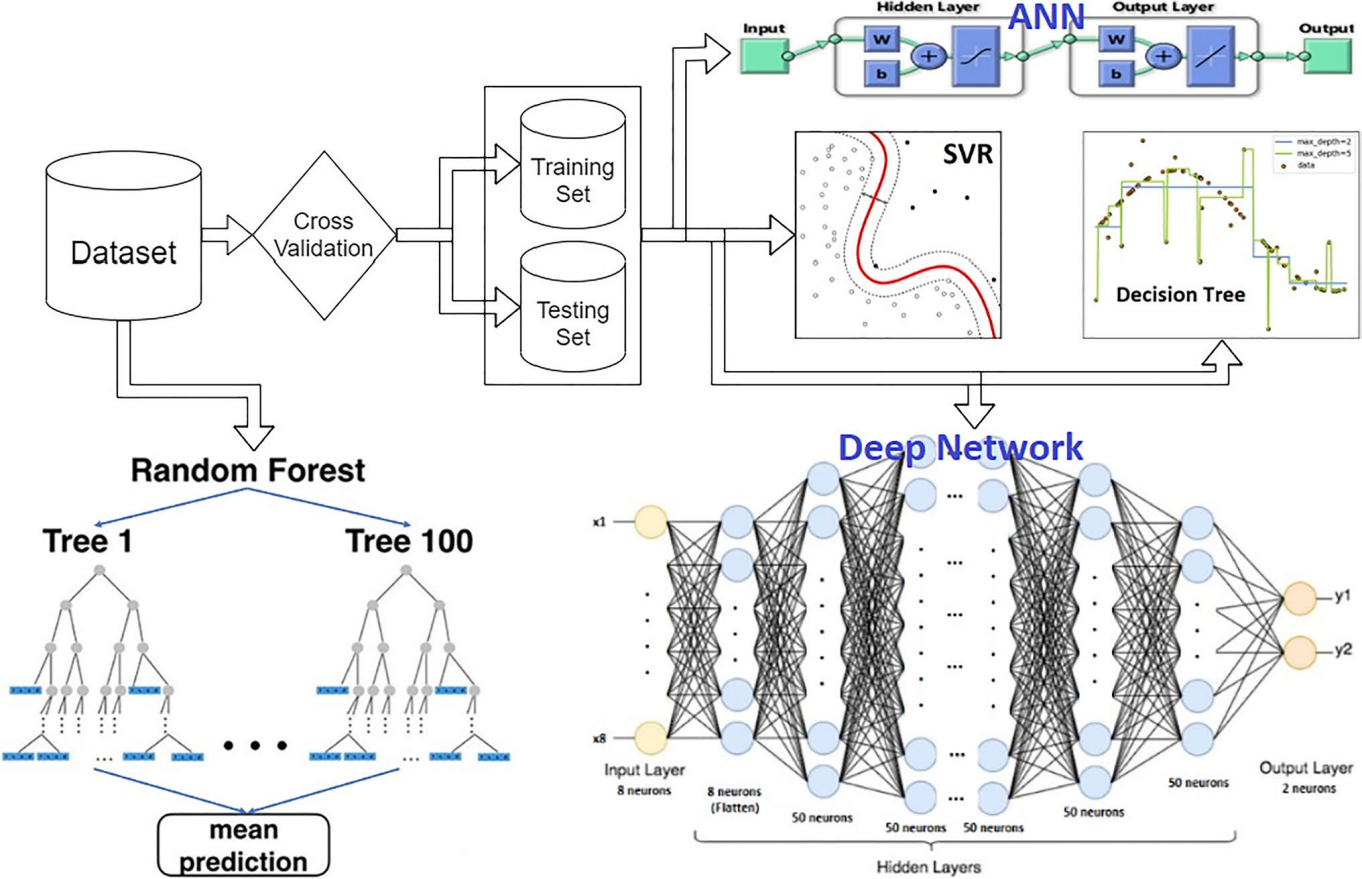
Flow diagram for the training procedure

The training and testing sets have been used to train and validate ANN, SVR, decision tree regressor (DTR), and deep neural network models. First, however, all the dataset has been used with the random forest regressor to obtain the percentage importance of each input feature. Figure 4 shows a flow diagram for the whole training process.

### Support vector regression

**Fig. 5:**
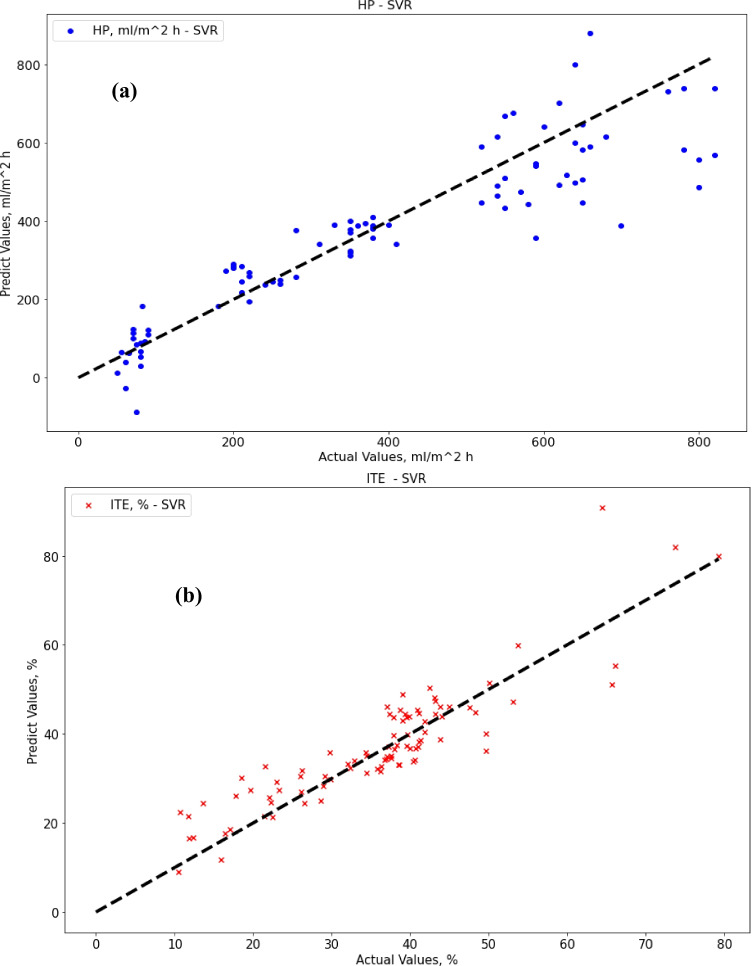
**a** Actual vs. predicted figure for HP output with SVR, **b** actual vs. predicted figure for ITE output with SVR

**Fig. 6:**
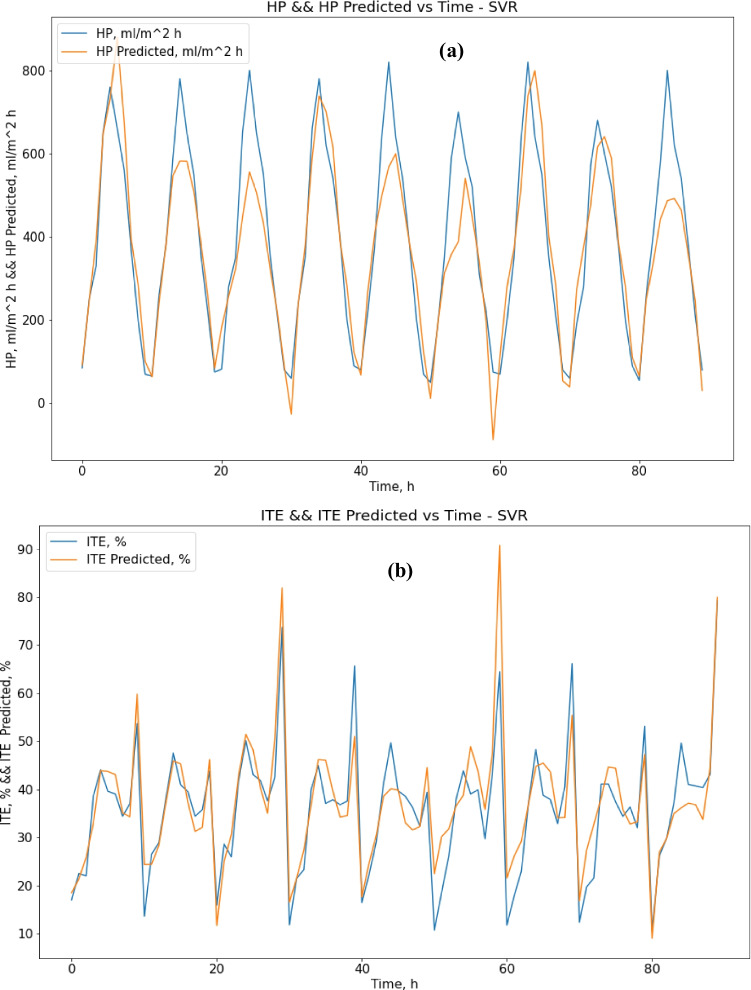
**a** Actual and predicted w.r.t. time for HP output with SVR, **b** actual and predicted w.r.t. time for ITE output with SVR

After proper training until convergence, the SVR gave good performance results for ITE output but not for HP output. Regarding SVR results, the MAE score was 70.35 (ml/m^2^ h) for HP output and 4.52% for ITE output. The R-squared score was 0.82 for HP output and 0.79 for ITE output, while the RMSE score was 98.23 (ml/m^2^ h) for HP output and 6.07% for ITE output. MRE score was 0.245 (ml/m^2^ h) for HP output and 0.155% for ITE output. Figure 5 a and b show actual vs. predicted for HP output and ITE output, respectively. Figure 6 a and b plot actual and predicted w.r.t time for HP and ITE outputs, respectively. It can be seen that the model gives a good prediction for the ITE output but not for the HP output. Hence, we will work on another model. Also, the R-squared values for both outputs are not good enough, 0.82 and 0.79.

### Decision tree regressor

**Fig. 7:**
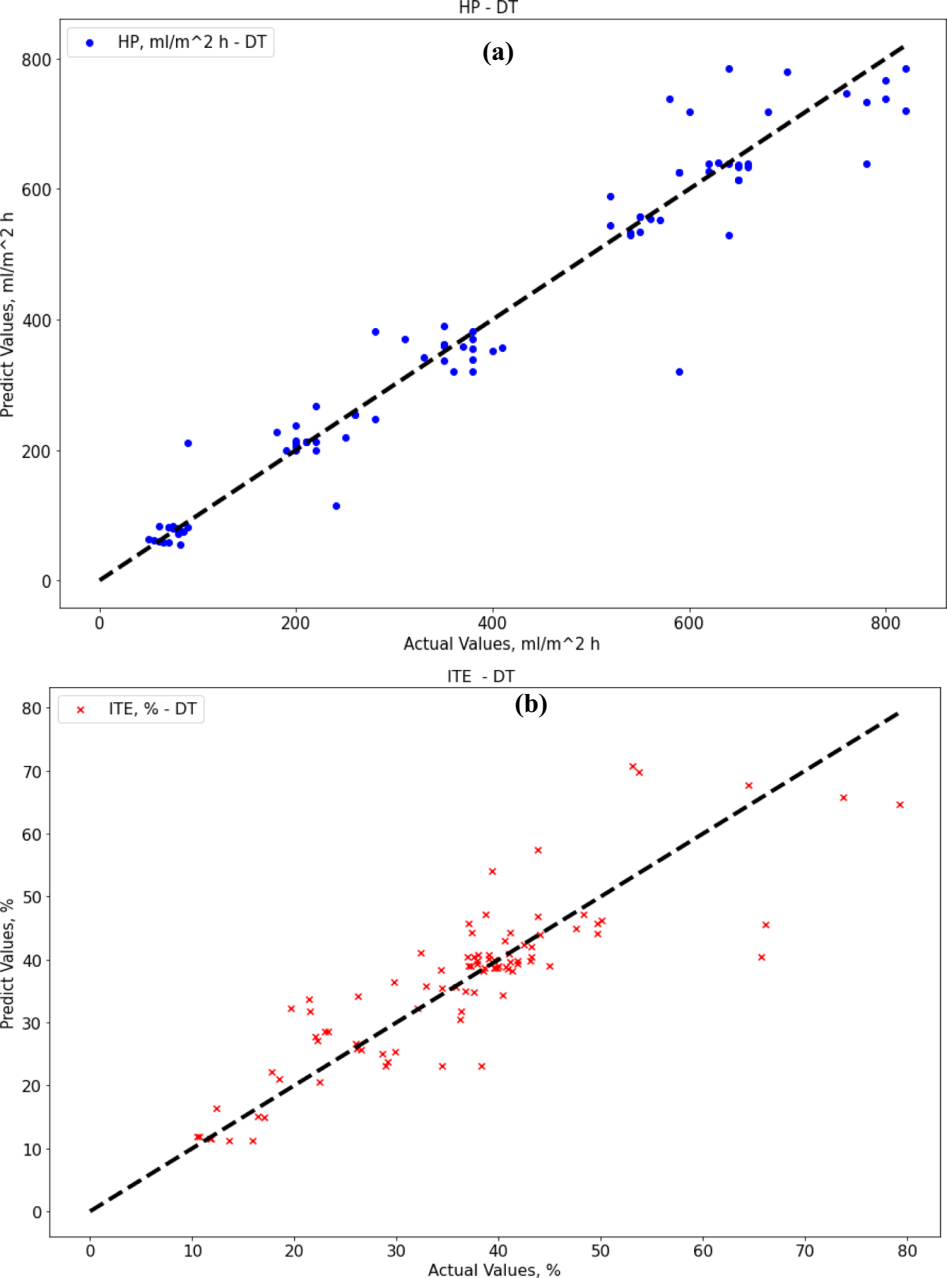
**a** Actual vs. predicted figure for HP output with DTR; **b** actual vs. predicted figure for ITE output with DTR

**Fig. 8:**
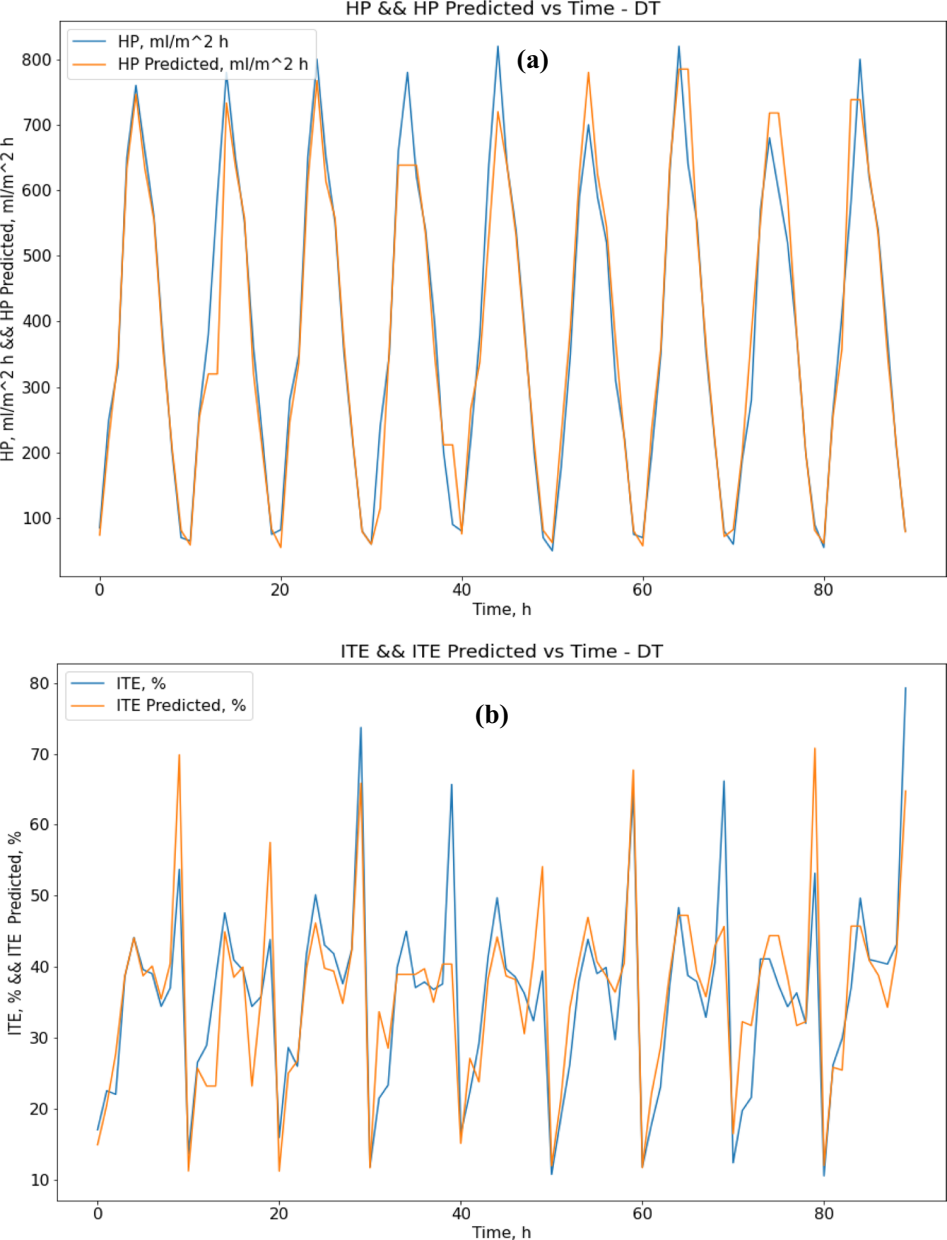
**a** Actual and predicted w.r.t. time for HP output with DTR; **b** actual and predicted w.r.t. time for ITE output with DTR

The decision tree algorithm gave better performance results than the SVR method. Regarding decision tree results, the MAE score was 33.67 (ml/m^2^ h) for HP output, and 4.63% for ITE output, which is better than SVR results only for HP output. The R-squared score was 0.94 for HP output and 0.74 for ITE output, while the RMSE score was 55.35 (ml/m^2^ h) for HP output and 6.76% for ITE output. MRE score was 0.109 (ml/m^2^ h) for HP output and 0.136% for ITE output. Figure 7a and b show actual vs. predicted HP output and ITE output, respectively. Figure 8a and b plot actual and predicted w.r.t time for HP and ITE outputs, respectively. It can be seen that the model gives a good prediction for the HP output but not for the ITE output. Hence, we will work on another model. Also, the R-squared values for ITE output are not good enough, 0.74.

### Artificial neural network

**Fig. 9:**
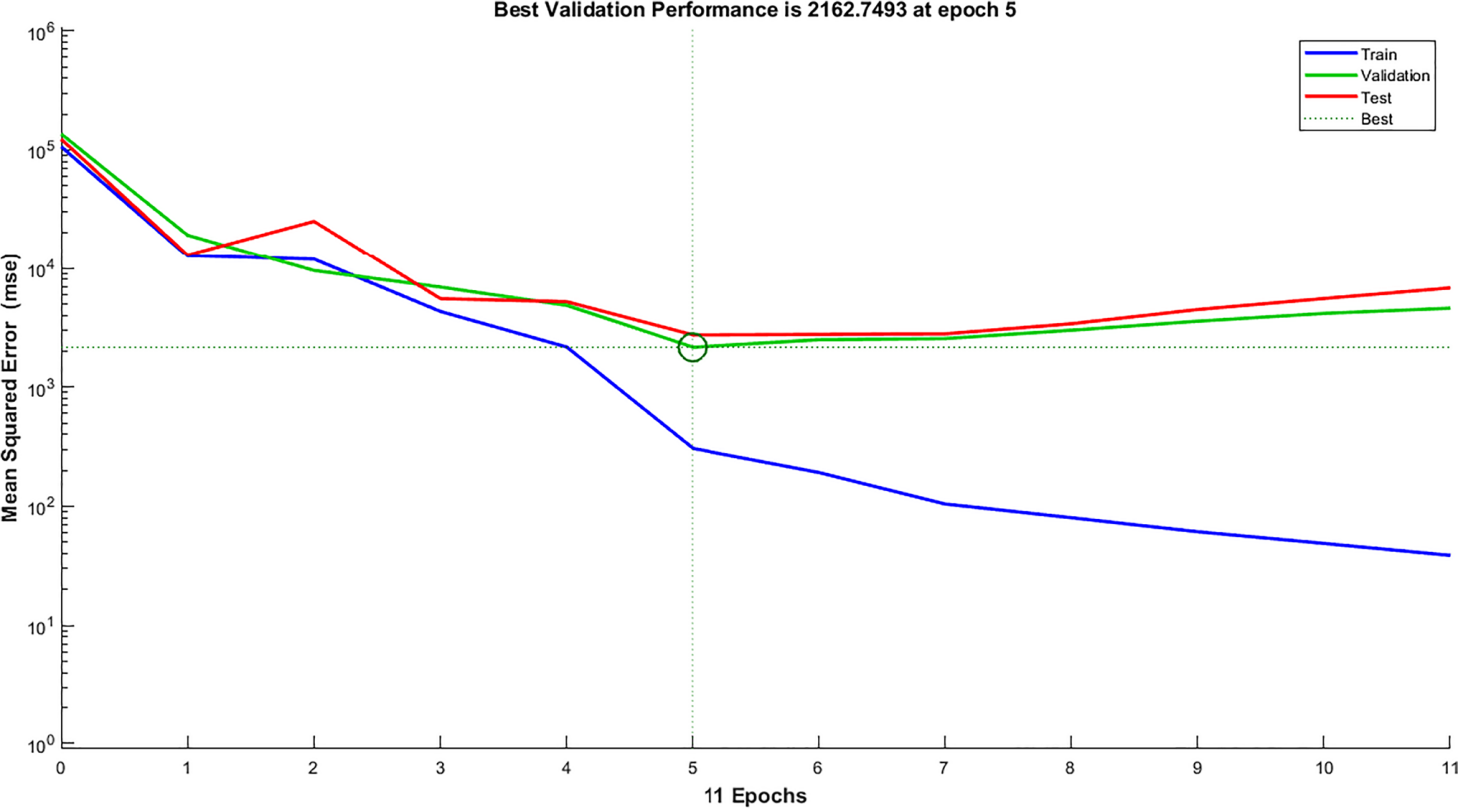
MATLAB training performance convergence plot

**Fig. 10:**
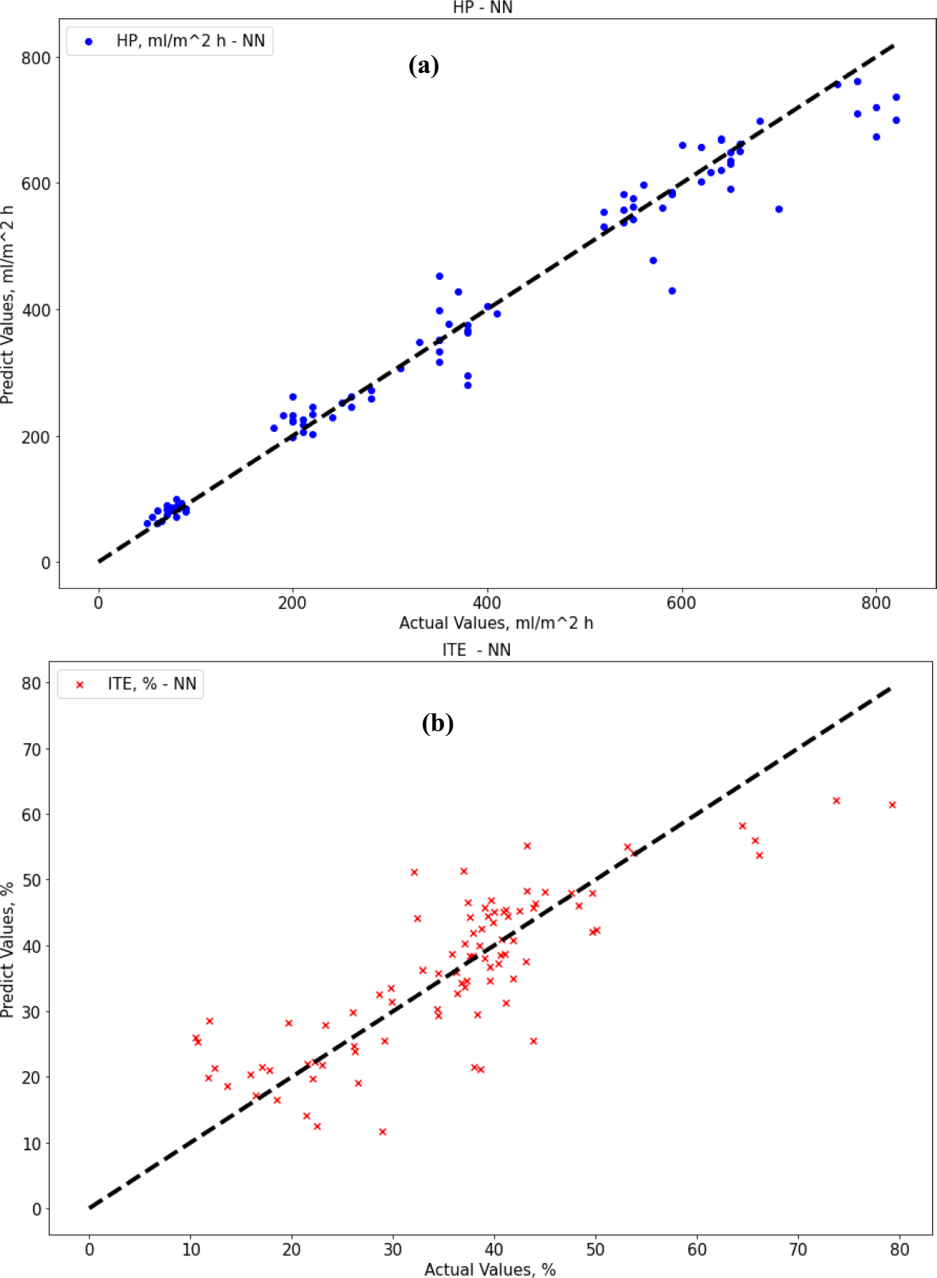
**a** Actual vs. predicted figure for HP output with ANN; **b** actual vs. predicted figure for ITE output with ANN

**Fig. 11:**
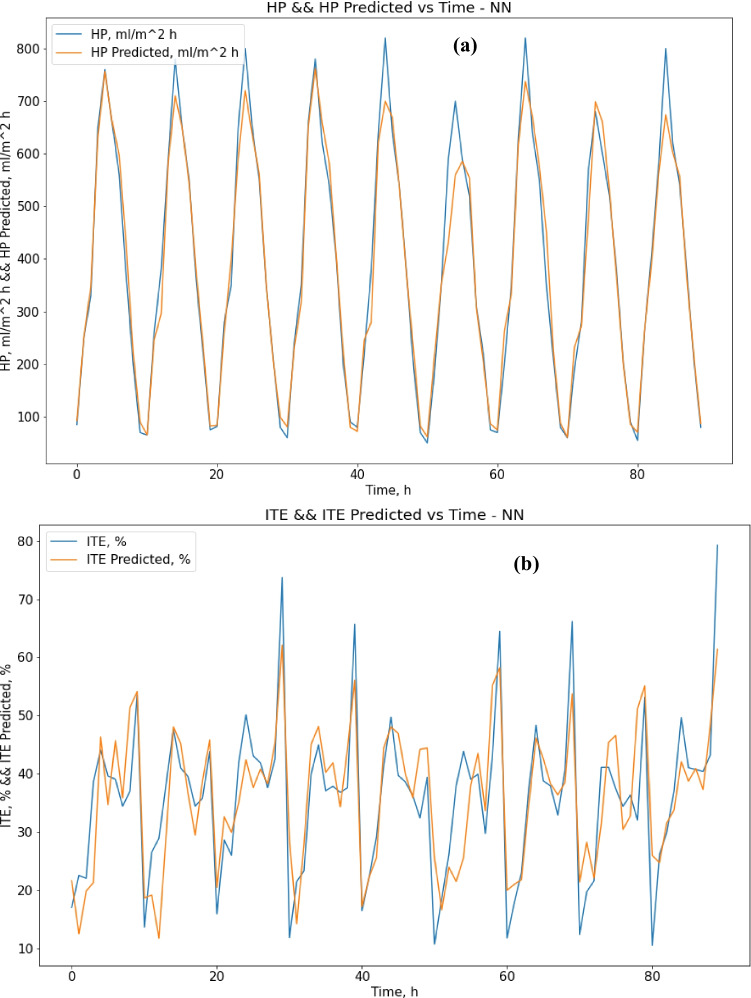
**a** Actual and predicted w.r.t. time for HP output with ANN; **b** actual and predicted w.r.t. time for ITE output with ANN

Regarding the ANN method, the results were not much different from decision tree results. Regarding ANN results, the MAE score was 27.98 (ml/m^2^ h) for HP output and 5.72% for ITE output, which is a bit better than decision tree results for HP output, and a bit worse for ITE output as expected [87]. The R-squared score was 0.965 for HP output and 0.676 for ITE output, while the RMSE score was 43.5 (ml/m^2^ h) for HP output and 7.54% for ITE output. MRE score was 0.087 (ml/m^2^ h) for HP output and 0.205% for ITE output. Figure 9 shows that the best validation performance is achieved at epoch five; after that, it starts to diverge away. Figure 10a and b show actual vs. predicted HP output and ITE output, respectively. Figure 11a and b plot actual and predicted w.r.t time for HP and ITE outputs, respectively. It can be seen that we need a better model.

### Deep neural network

**Fig. 12:**
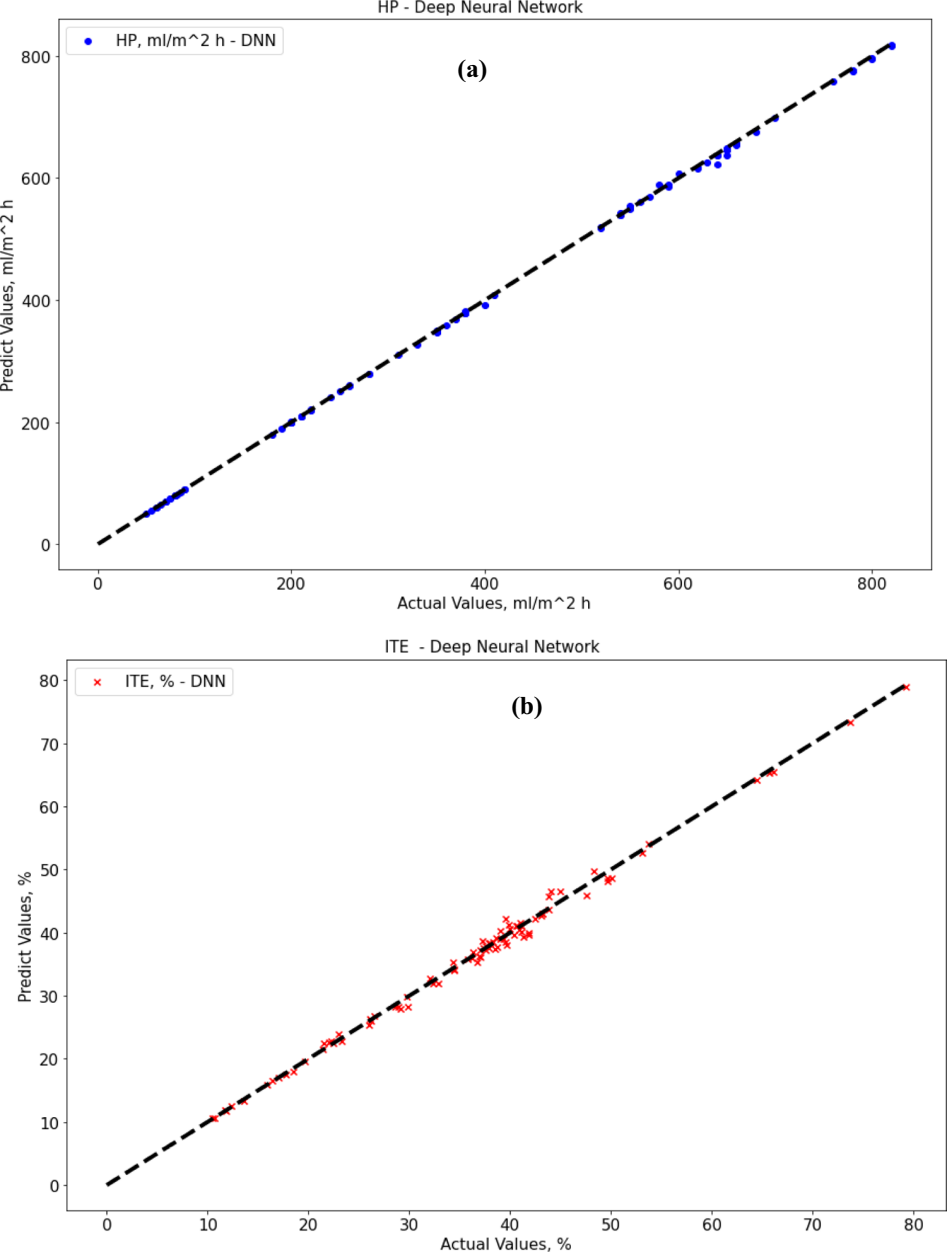
**a** Actual vs. predicted figure for HP output with DNN; **b** actual vs. predicted figure for ITE output with DNN

**Fig. 13:**
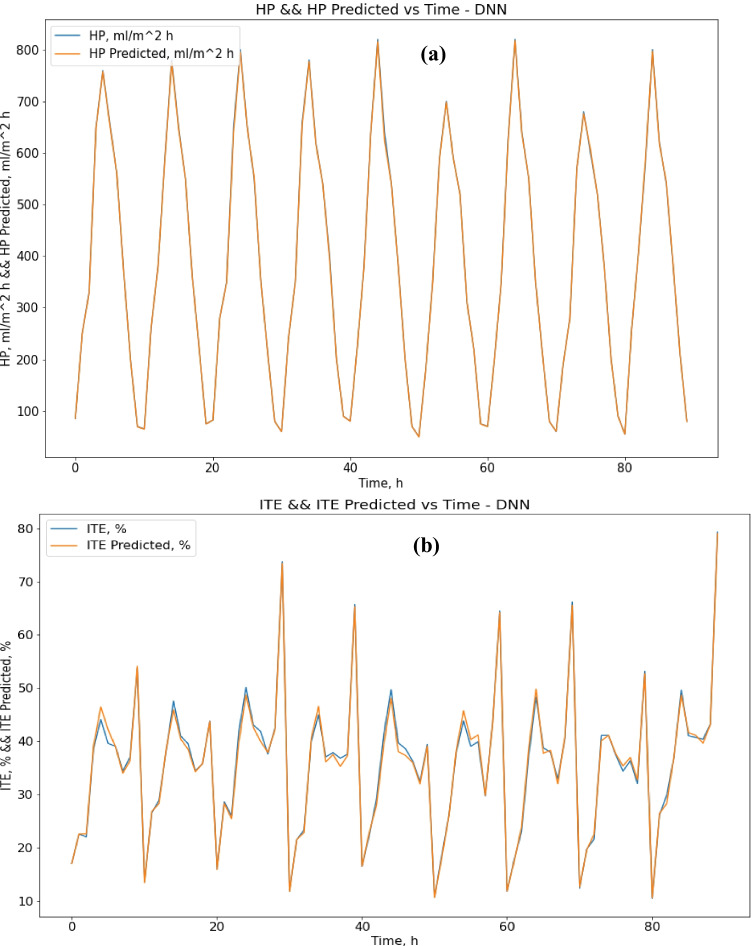
**a** Actual and predicted w.r.t. time for HP output with DNN; **b** actual and predicted w.r.t. time for ITE output with DNN

The deep neural network gave the best performance results within all tested algorithms, including SVR, decision tree, and neural network. regarding deep neural network results; the MAE score was 1.94 for HP output and 0.67 for ITE output, which is much better than neural network, decision tree, and SVR results for both HP and ITE output. The R-squared score was 0.9998 for HP output and 0.995 for ITE output which is a great result, while the RMSE score was 3.3 for HP output and 0.9 for ITE output. MRE score was 0.0047 for HP output and 0.0185 for ITE output. Figure 12a and b show actual vs. predicted HP output and ITE output, respectively. Figure 13a and b plot actual and predicted w.r.t time for HP and ITE outputs, respectively. It can be seen that HP predicted output is almost the same as the actual output with almost no error. Obviously, actual and predicted w.r.t time have a complete match for the HP output and very close for the ITE output.

### Comparison between SVR, decision tree, and deep neural network

**Fig. 14:**
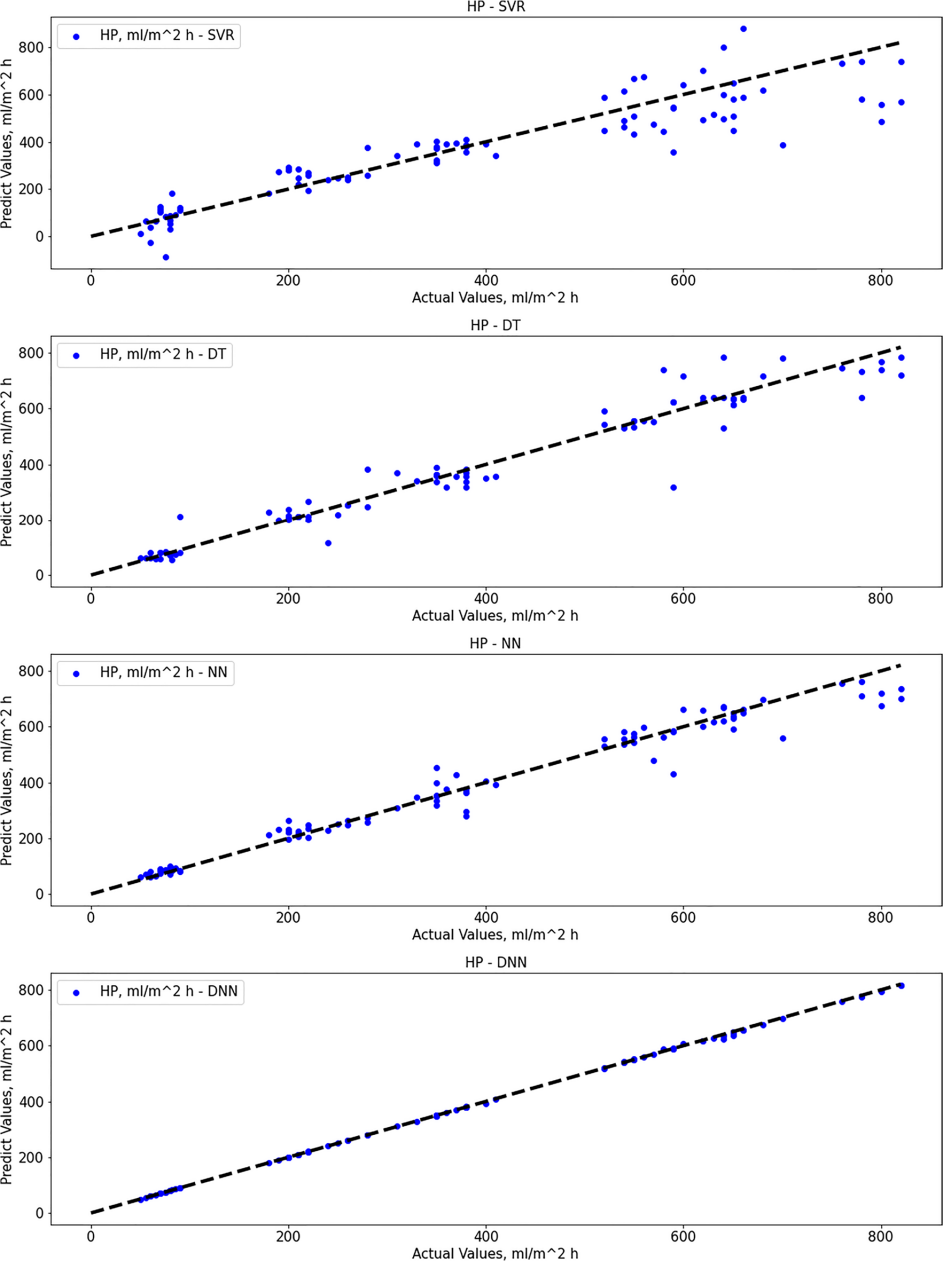
Actual vs. predicted for HP output (SVR vs. DT vs. NN vs. DNN)

**Fig. 15:**
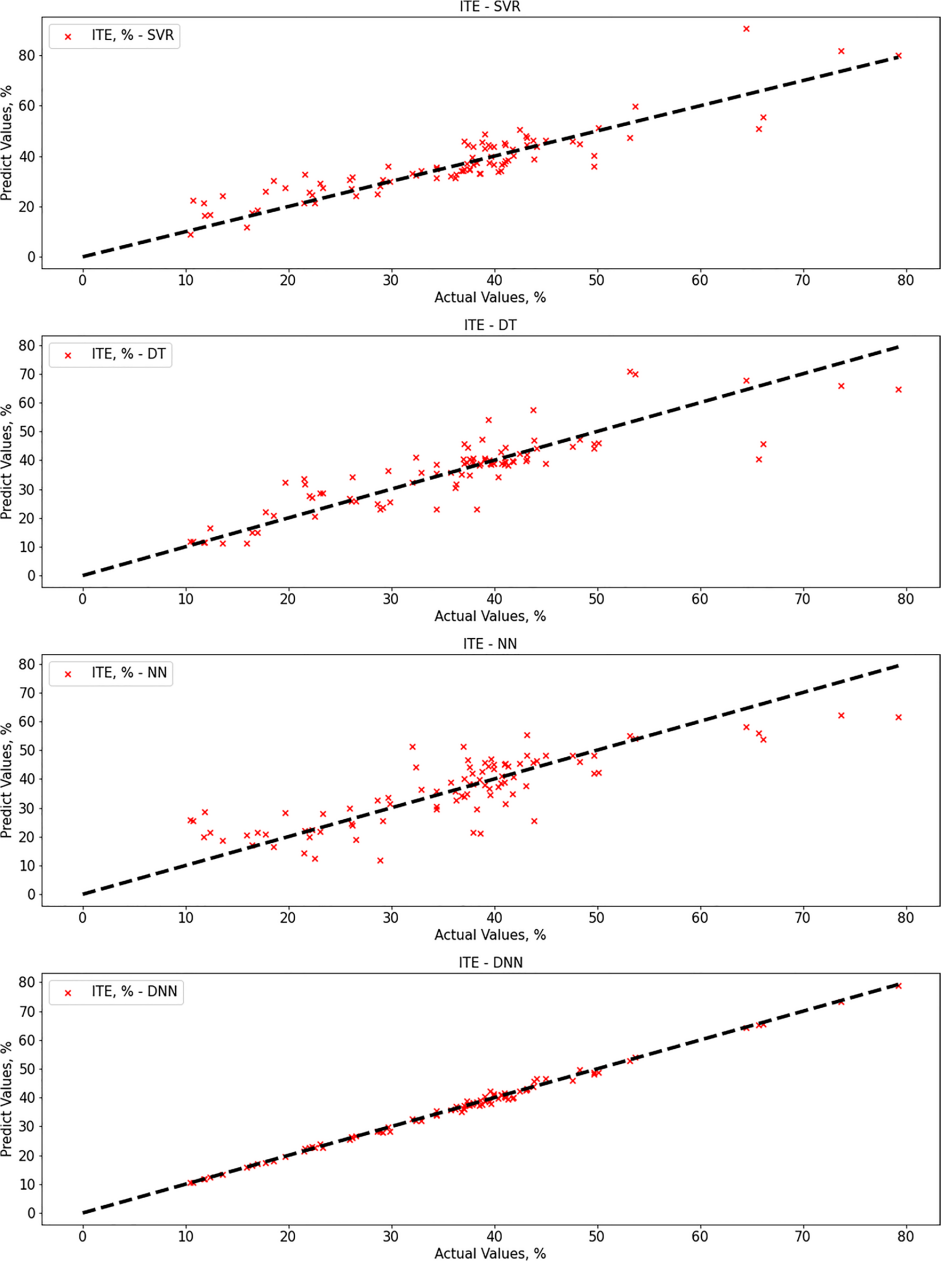
Actual vs. predicted for ITE output (SVR vs. DT vs. NN vs. DNN)

Figures 14 and 15 show a plot of comparison between actual vs. predicted HP output and ITE output, respectively, for four models: SVR, decision tree, neural network, and deep neural network. First, the deep neural network gives the best prediction results, then the neural network, then the decision tree, then SVR for the HP output. After that, however, the deep neural network gives the best prediction results for the ITE output, SVR, decision tree, and neural network.

Table 3 compares SVR, decision tree, neural network, and deep neural network using R-squared, RMSE, MAE, and MRE scores beside mean and Std.

**Table 3:** R-squared, RMS, MAE, and MRE (SVR vs DT vs NN vs DNN)

	R-squared	RMSE	MAE	MRE	Mean	Std
SVR (HP) (ml/m^2^ h)	0.82	98.23	70.35	0.245	366.96	208.45
SVR (ITE) (%)	0.79	6.07	4.52	0.155	37.06	13.08
Decision tree (HP) (ml/m^2^ h)	0.94	55.35	33.67	0.109	383.66	230.59
Decision tree (ITE) (%)	0.74	6.76	4.63	0.136	36.19	12.7
Neural network (HP) (ml/m^2^ h)	0.965	43.5	27.98	0.087	381.59	221.05
Neural network (ITE) (%)	0.676	7.54	5.72	0.205	36.03	11.95
Deep neural network (HP) (ml/m^2^ h)	0.9998	3.3	1.94	0.0047	385.05	232.6
Deep neural network (ITE) (%)	0.9953	0.9	0.67	0.0185	35.83	13.19

### Feature selection

**Fig. 16:**
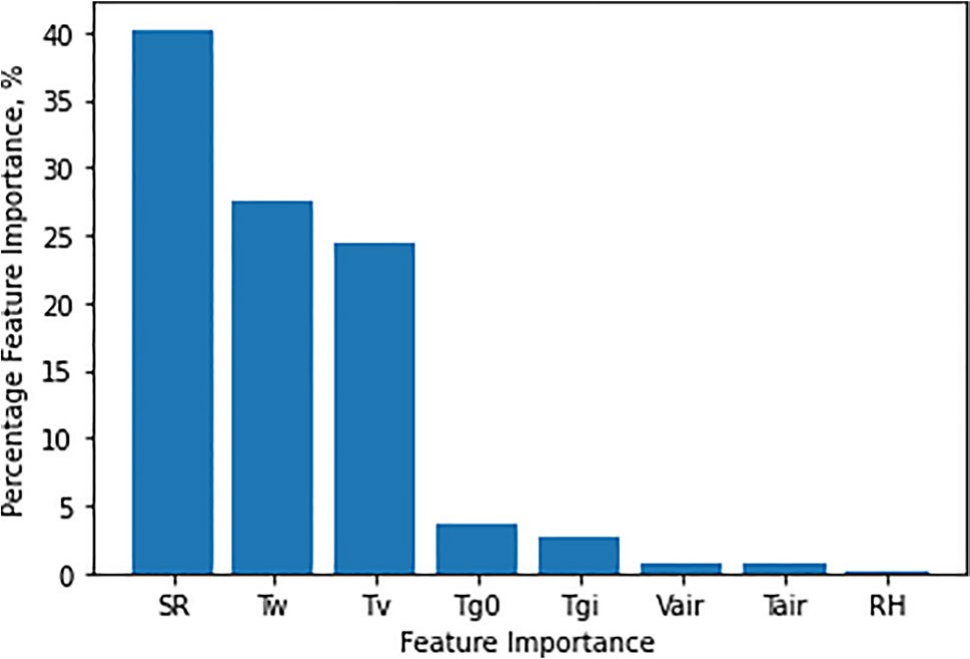
Percentage importance for each input feature

The arrangement of the features’ importance parameters had been in decreasing logical sequence. From straight to indirect impact on productivity, the percentage importance of each input characteristic had been calculated, according to the data displayed in Fig. 16. The most important parameters were SR and *T*_w_ by about 40.22% and 27.62% because they directly influenced the rate of evaporation and, consequently, the freshwater production. Also, the vapor had a feature importance of about 24.34% followed by the glass out and inlet, which had 3.5 and 2.6% respectively. Additionally, the air speed and air temperature had the same feature importance values of about 0.75 and 0.7% respectively. Finally, the lower feature importance was related to a relative humidity of 0.18%.

## Conclusions

The effects of employing a stepped double-slope solar still (SDSSS) with a cotton wick and cobalt oxide (Co_3_O_4_) nanofluid with 1wt% on its steps are shown in this research. Every hour from 9:00 to 17:00 (GMT + 2), the results were measured and repeated for 9 days. Furthermore, four different machine learning models: support vector regressor (SVR), decision tree, neural network, and deep neural network. The results showed that the neural network gave the worst results, especially for validation and test data, which means it failed to generalize. Hence, the neural network is almost out of comparison.

- The results demonstrated that the deep neural network gives the best results for both outputs, which can be verified from comparison plots and the values of R-squared, RMS, MAE, and MRE scores, which outweigh the favor of the deep neural network with big differences.
- The next best model depends on which output we consider since regarding HP output, it can be seen that the neural network is the next best model, then the decision tree comes after it. However, regarding the ITE output, it can be seen that the next best model is the SVR then the decision tree comes after it.
- Regarding the feature importance, it can be noted that the most important input feature is SR, Tw, and Tv.

## Data Availability

Not applicable.

## Abbreviations

*ANN*: Artificial neural network
*HP*: Hourly productivity
*SDSSS*: Stepped double-slope solar still
*SS*: Solar still
*RMSE*: Root mean square error
*R*: Regression
*R-squared*: Coefficient of determination
*MRE*: Mean relative error
*MAE*: Mean absolute error
*COV*: Coefficient of variance
*EC*: Efficiency coefficient
*OI*: Overall index of model performance
*CRM*: Coefficient of residual mass
